# Trichloridotris{*N*-[phen­yl(pyridin-2-yl)methyl­idene]hydroxyl­amine-κ^2^ *N*,*N*′}neodymium(III)

**DOI:** 10.1107/S1600536812014055

**Published:** 2012-04-13

**Authors:** Hua Yang

**Affiliations:** a College of Chemistry and Chemical Engineering, Yan’an University, Yan’an, Shaanxi 716000, People’s Republic of China

## Abstract

In the title compound, [NdCl_3_(C_12_H_10_N_2_O)_3_], the central Nd^III^ ion is nine-coordinated by six N atoms from three bidentate chelate *N*-[phen­yl(pyridin-2-yl)methyl­idene]hydroxyl­amine ligands and three Cl^−^ ions, and displays a distorted tricapped trigonal prismatic geometry. The complex mol­ecules are stabilized by intra­molecular O—H⋯Cl hydrogen bonds.

## Related literature

For complexes of oximes, see: Kukushkin & Pombeiro (1999 ▶); Milios *et al.* (2007 ▶); Fritsky *et al.* (2004 ▶); Xu *et al.* (2007 ▶); Papatriantafyllopoulou *et al.* (2009 ▶). For 3*d*-metal complexes of *N*-[phen­yl(pyridine-2-yl)methyl­idene]hydroxyl­amine, see: Milios *et al.* (2003 ▶); Milios *et al.* (2004 ▶). For an Sm complex with this ligand, see: Lei *et al.* (2012 ▶).
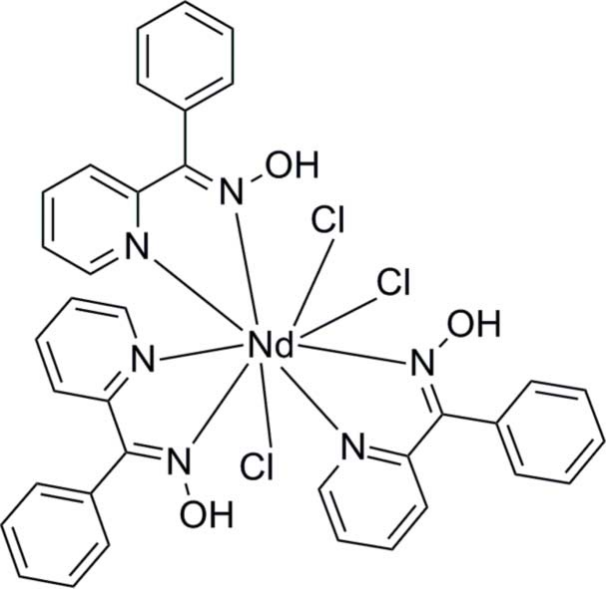


## Experimental

### 

#### Crystal data

[NdCl_3_(C_12_H_10_N_2_O)_3_]
*M* *_r_* = 845.25
Triclinic, 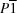

*a* = 8.6367 (17) Å
*b* = 10.460 (2) Å
*c* = 19.847 (4) Å
α = 91.87 (3)°
β = 94.38 (3)°
γ = 92.80 (3)°
*V* = 1784.4 (6) Å^3^
*Z* = 2
Mo *K*α radiation
μ = 1.72 mm^−1^
*T* = 293 K
0.31 × 0.18 × 0.13 mm

#### Data collection

Bruker SMART CCD-detector diffractometer
Absorption correction: multi-scan (*SADABS*; Bruker, 2000 ▶) *T*_min_ = 0.617, *T*_max_ = 0.807
30524 measured reflections
7775 independent reflections
7155 reflections with *I* > 2σ(*I*)
*R*_int_ = 0.028

#### Refinement

*R*[*F* ^2^ > 2σ(*F* ^2^)] = 0.022
*wR*(*F* ^2^) = 0.059
*S* = 1.04
7775 reflections
445 parameters
H-atom parameters constrained
Δρ_max_ = 0.50 e Å^−3^
Δρ_min_ = −0.44 e Å^−3^

### 

Data collection: *SMART* (Bruker, 2000 ▶); cell refinement: *SAINT* (Bruker, 2000 ▶); data reduction: *SAINT*; program(s) used to solve structure: *SHELXS97* (Sheldrick, 2008 ▶); program(s) used to refine structure: *SHELXL97* (Sheldrick, 2008 ▶); molecular graphics: *SHELXTL* (Sheldrick, 2008 ▶); software used to prepare material for publication: *SHELXTL*.

## Supplementary Material

Crystal structure: contains datablock(s) I, global. DOI: 10.1107/S1600536812014055/zs2190sup1.cif

Structure factors: contains datablock(s) I. DOI: 10.1107/S1600536812014055/zs2190Isup2.hkl

Additional supplementary materials:  crystallographic information; 3D view; checkCIF report

## Figures and Tables

**Table 1:** Selected bond lengths (Å)

Nd1—N2	2.604 (2)
Nd1—N1	2.661 (2)
Nd1—N5	2.680 (2)
Nd1—N4	2.6953 (19)
Nd1—N6	2.7018 (19)
Nd1—N3	2.742 (2)
Nd1—Cl3	2.7686 (8)
Nd1—Cl2	2.7903 (9)
Nd1—Cl1	2.8296 (10)

**Table 2:** Hydrogen-bond geometry (Å, °)

*D*—H⋯*A*	*D*—H	H⋯*A*	*D*⋯*A*	*D*—H⋯*A*
O1—H1*A*⋯Cl3	0.82	2.22	2.966 (2)	152
O2—H2*A*⋯Cl1	0.82	2.19	2.9290 (19)	151
O3—H3*A*⋯Cl2	0.82	2.19	2.930 (2)	150

**Fig. 1.**
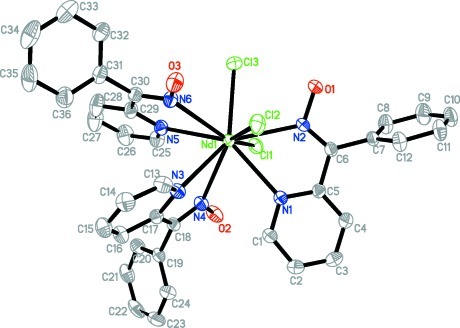
The molecular structure of the title complex, showing atom labels and 30% probability displacement ellipsoids.

**Fig. 2.**
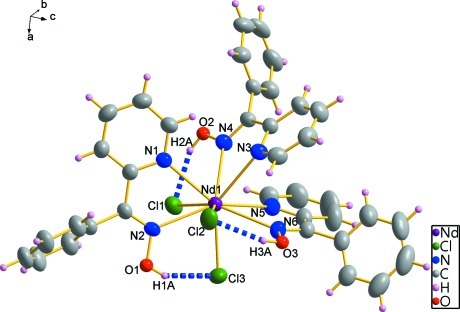
Intramolecular hydrogen-bonding interactions in the title complex, with hydrogen bonds shown as dashed lines.

## supplementary crystallographic information

### Comment

The coordination chemistry of oximes (Kukushkin & Pombeiro, 1999; Milios *et al.*, 2007) continues to attract considerable attention, with the efforts of several research groups driven by a number of considerations. These include the use of metal oxime complexes in supramolecular chemistry (Fritsky *et al.*, 2004) and the employment of oximate ligands in the synthesis of complexes with interesting magnetic properties (Xu *et al.*, 2007; Papatriantafyllopoulou *et al.*, 2009; Milios *et al.*, 2007). *N*-[phenyl(pyridine-2-yl)methylidene]hydroxylamine [(py)C(ph)NOH], is one of the oximes that is currently a popular ligand for synthesis of the 3*d*-metal complexes (Milios *et al.*, 2003; Milios *et al.*, 2004). However, the structures of rare earth metal complexes with this ligand are uncommon in the crystallographic literature. Here we report the structure of the neodymium complex with [(py)C(ph)NOH], the title compound [NdCl_3_(C_12_H_10_N_2_O)_3_], which was synthesized by the reaction of NdCl_3_ . 6H_2_O with the ligand under autogenous pressure. The title compound is isomorphous with the Sm^III^ analogue (Lei *et al.*, 2012).

In the title complex, the central Nd^III^ ion is nine-coordinated by six nitrogen atoms from three bidentate chelate ligands and three Cl^-^ ions [Nd—N range, 2.604 (2)–2.742 (2) Å; Nd—Cl, 2.7686 (8)–2.8296 (10) Å (Table 1)] and displays a distorted tricapped trigonal prismatic geometry (Fig. 1). The discrete complex molecules are stabilized by intramolecular O—H···Cl hydrogen bonds (Table 2, Fig. 2).

### Experimental

A mixture of phenyl-2-pyridyl ketone oxime (0.0198 g, 0.10 mmol), NdCl_3_ . 6H_2_O (0.0179 g, 0.05 mmol), and ethanol (2 mL) was sealed in a 6 mL Pyrex tube. The tube was heated at 80 °C for 4 days under autogenous pressure. Cooling of the resultant solution to room temperature gave colourless crystals of the product. The crystals were collected by filtration, washed with ethanol (2 mL) and dried in air. Yield: 54%. Anal. Calcd. for C_36_H_30_Cl_3_N_6_NdO_3_: C, 51.15; H, 3.58; N, 9.94%. Found: C, 50.93; H, 3.43; N, 9.76%.

### Refinement

H atoms were placed in calculated positions and included in the refinement using a riding-model approximation, with C—H = 0.93 Å and O—H = 0.82 Å, and with *U*_iso_(H) = 1.2*U*_eq_(C) or 1.5*U*_eq_(O).

### Crystal data

**Table:**

[NdCl_3_(C_12_H_10_N_2_O)_3_]	*Z* = 2
*M**_r_* = 845.25	*F*(000) = 846
Triclinic, *P*1	char
Hall symbol: -P 1	*D*_x_ = 1.573 Mg m^−^^3^
*a* = 8.6367 (17) Å	Mo *K*α radiation, λ = 0.71073 Å
*b* = 10.460 (2) Å	Cell parameters from 7737 reflections
*c* = 19.847 (4) Å	θ = 2.2–27.0°
α = 91.87 (3)°	µ = 1.72 mm^−^^1^
β = 94.38 (3)°	*T* = 293 K
γ = 92.80 (3)°	Block, colourless
*V* = 1784.4 (6) Å^3^	0.31 × 0.18 × 0.13 mm

### Data collection

**Table:**

Bruker SMART CCD-detector diffractometer	7775 independent reflections
Radiation source: fine-focus sealed tube	7155 reflections with *I* > 2σ(*I*)
Graphite monochromator	*R*_int_ = 0.028
φ and ω scans	θ_max_ = 27.0°, θ_min_ = 1.0°
Absorption correction: multi-scan (*SADABS*; Bruker, 2000)	*h* = −11→11
*T*_min_ = 0.617, *T*_max_ = 0.807	*k* = −13→13
30524 measured reflections	*l* = −25→25

### Refinement

**Table:**

Refinement on *F*^2^	Primary atom site location: structure-invariant direct methods
Least-squares matrix: full	Secondary atom site location: difference Fourier map
*R*[*F*^2^ > 2σ(*F*^2^)] = 0.022	Hydrogen site location: inferred from neighbouring sites
*wR*(*F*^2^) = 0.059	H-atom parameters constrained
*S* = 1.04	*w* = 1/[σ^2^(*F*_o_^2^) + (0.0316*P*)^2^ + 0.3895*P*] where *P* = (*F*_o_^2^ + 2*F*_c_^2^)/3
7775 reflections	(Δ/σ)_max_ = 0.002
445 parameters	Δρ_max_ = 0.50 e Å^−^^3^
0 restraints	Δρ_min_ = −0.44 e Å^−^^3^

### Special details

**Table:**

Geometry. All e.s.d.'s (except the e.s.d. in the dihedral angle between two l.s. planes) are estimated using the full covariance matrix. The cell e.s.d.'s are taken into account individually in the estimation of e.s.d.'s in distances, angles and torsion angles; correlations between e.s.d.'s in cell parameters are only used when they are defined by crystal symmetry. An approximate (isotropic) treatment of cell e.s.d.'s is used for estimating e.s.d.'s involving l.s. planes.
Refinement. Refinement of *F*^2^ against ALL reflections. The weighted *R*-factor *wR* and goodness of fit *S* are based on *F*^2^, conventional *R*-factors *R* are based on *F*, with *F* set to zero for negative *F*^2^. The threshold expression of *F*^2^ > σ(*F*^2^) is used only for calculating *R*-factors(gt) *etc*. and is not relevant to the choice of reflections for refinement. *R*-factors based on *F*^2^ are statistically about twice as large as those based on *F*, and *R*- factors based on ALL data will be even larger.

### Fractional atomic coordinates and isotropic or equivalent isotropic displacement parameters (Å^2^)

**Table:**

	*x*	*y*	*z*	*U*_iso_*/*U*_eq_	
Nd1	0.170030 (12)	0.594157 (10)	0.248609 (5)	0.03370 (4)	
Cl2	0.10720 (8)	0.35456 (5)	0.30113 (3)	0.05379 (15)	
Cl1	0.27975 (7)	0.70288 (6)	0.13140 (3)	0.05048 (14)	
Cl3	0.48680 (7)	0.57109 (6)	0.27455 (3)	0.05349 (15)	
N2	0.2436 (2)	0.41331 (18)	0.16542 (10)	0.0429 (4)	
N6	0.2375 (2)	0.61037 (18)	0.38390 (9)	0.0437 (4)	
N1	−0.0455 (2)	0.48814 (17)	0.15808 (9)	0.0397 (4)	
N3	−0.0819 (2)	0.65372 (18)	0.31740 (9)	0.0415 (4)	
N5	0.2564 (2)	0.81945 (17)	0.31196 (10)	0.0434 (4)	
C27	0.3334 (5)	1.0579 (3)	0.37346 (19)	0.0884 (11)	
H27	0.3572	1.1381	0.3943	0.106*	
N4	−0.0185 (2)	0.77901 (18)	0.20749 (9)	0.0418 (4)	
C7	0.2007 (3)	0.2515 (2)	0.07372 (11)	0.0416 (5)	
C18	−0.1209 (2)	0.8276 (2)	0.24228 (11)	0.0378 (4)	
O1	0.3882 (2)	0.36271 (19)	0.17316 (10)	0.0627 (5)	
H1A	0.4419	0.4034	0.2031	0.094*	
O2	−0.0065 (2)	0.83670 (19)	0.14624 (9)	0.0592 (5)	
H2A	0.0687	0.8104	0.1282	0.089*	
C19	−0.2129 (3)	0.9365 (2)	0.21929 (11)	0.0397 (5)	
C5	−0.0047 (3)	0.4056 (2)	0.10935 (11)	0.0394 (5)	
O3	0.2290 (2)	0.50316 (16)	0.42307 (8)	0.0576 (4)	
H3A	0.2028	0.4398	0.3987	0.086*	
C17	−0.1480 (2)	0.7670 (2)	0.30694 (11)	0.0384 (5)	
C13	−0.1152 (3)	0.5941 (2)	0.37345 (13)	0.0502 (6)	
H13	−0.0759	0.5139	0.3803	0.060*	
C31	0.2881 (3)	0.7204 (3)	0.49380 (12)	0.0508 (6)	
C6	0.1547 (3)	0.3581 (2)	0.11766 (11)	0.0399 (5)	
C21	−0.2296 (4)	1.1591 (3)	0.19732 (14)	0.0624 (7)	
H21	−0.1853	1.2421	0.1996	0.075*	
C1	−0.1928 (3)	0.5235 (2)	0.15376 (12)	0.0459 (5)	
H1	−0.2241	0.5772	0.1879	0.055*	
C4	−0.1058 (3)	0.3652 (3)	0.05469 (12)	0.0521 (6)	
H4	−0.0734	0.3112	0.0210	0.063*	
C30	0.2678 (3)	0.7144 (2)	0.41872 (11)	0.0440 (5)	
C24	−0.3642 (3)	0.9139 (3)	0.19283 (13)	0.0512 (6)	
H24	−0.4110	0.8319	0.1923	0.061*	
C14	−0.2040 (3)	0.6440 (3)	0.42155 (13)	0.0569 (6)	
H14	−0.2222	0.5994	0.4602	0.068*	
C25	0.2728 (3)	0.9250 (2)	0.27624 (14)	0.0537 (6)	
H25	0.2582	0.9167	0.2294	0.064*	
C20	−0.1452 (3)	1.0601 (2)	0.22234 (13)	0.0516 (6)	
H20	−0.0437	1.0759	0.2411	0.062*	
C29	0.2820 (3)	0.8323 (2)	0.37946 (12)	0.0475 (5)	
C23	−0.4461 (3)	1.0143 (3)	0.16705 (14)	0.0630 (7)	
H23	−0.5476	0.9994	0.1482	0.076*	
C32	0.4105 (3)	0.6620 (3)	0.52790 (13)	0.0559 (6)	
H32	0.4837	0.6224	0.5036	0.067*	
C16	−0.2384 (3)	0.8229 (2)	0.35265 (13)	0.0516 (6)	
H16	−0.2810	0.9013	0.3441	0.062*	
C33	0.4240 (4)	0.6624 (3)	0.59736 (15)	0.0744 (9)	
H33	0.5055	0.6222	0.6199	0.089*	
C11	0.1861 (4)	0.0299 (3)	0.03858 (14)	0.0635 (7)	
H11	0.1448	−0.0530	0.0427	0.076*	
C12	0.1387 (3)	0.1293 (2)	0.07905 (13)	0.0549 (6)	
H12	0.0648	0.1129	0.1098	0.066*	
C2	−0.3004 (3)	0.4845 (3)	0.10144 (14)	0.0554 (6)	
H2	−0.4018	0.5110	0.1006	0.067*	
C22	−0.3780 (4)	1.1357 (3)	0.16916 (14)	0.0676 (8)	
H22	−0.4332	1.2026	0.1513	0.081*	
C8	0.3121 (3)	0.2733 (3)	0.02820 (14)	0.0605 (7)	
H8	0.3573	0.3552	0.0250	0.073*	
C9	0.3564 (4)	0.1743 (3)	−0.01235 (15)	0.0738 (9)	
H9	0.4298	0.1897	−0.0434	0.089*	
C3	−0.2561 (3)	0.4062 (3)	0.05076 (14)	0.0612 (7)	
H3	−0.3258	0.3808	0.0142	0.073*	
C15	−0.2652 (3)	0.7611 (3)	0.41137 (14)	0.0597 (7)	
H15	−0.3238	0.7982	0.4435	0.072*	
C35	0.1970 (5)	0.7814 (4)	0.60097 (17)	0.0867 (11)	
H35	0.1257	0.8220	0.6260	0.104*	
C26	0.3100 (4)	1.0446 (3)	0.30484 (18)	0.0729 (8)	
H26	0.3191	1.1153	0.2780	0.087*	
C10	0.2927 (4)	0.0537 (3)	−0.00695 (14)	0.0663 (8)	
H10	0.3223	−0.0128	−0.0346	0.080*	
C36	0.1811 (4)	0.7812 (3)	0.53085 (15)	0.0708 (8)	
H36	0.0992	0.8216	0.5088	0.085*	
C34	0.3174 (5)	0.7219 (4)	0.63336 (16)	0.0888 (12)	
H34	0.3270	0.7220	0.6803	0.107*	
C28	0.3211 (4)	0.9497 (3)	0.41152 (16)	0.0735 (9)	
H28	0.3391	0.9562	0.4583	0.088*	

### Atomic displacement parameters (Å^2^)

**Table:**

	*U*^11^	*U*^22^	*U*^33^	*U*^12^	*U*^13^	*U*^23^
Nd1	0.03692 (7)	0.02906 (7)	0.03506 (7)	0.00424 (4)	0.00134 (4)	−0.00019 (4)
Cl2	0.0757 (4)	0.0322 (3)	0.0531 (3)	−0.0011 (3)	0.0043 (3)	0.0023 (2)
Cl1	0.0546 (3)	0.0522 (3)	0.0471 (3)	0.0083 (3)	0.0152 (3)	0.0057 (3)
Cl3	0.0391 (3)	0.0638 (4)	0.0559 (3)	0.0057 (3)	−0.0057 (2)	−0.0067 (3)
N2	0.0381 (9)	0.0402 (10)	0.0505 (11)	0.0122 (8)	0.0013 (8)	−0.0055 (8)
N6	0.0537 (11)	0.0375 (10)	0.0399 (10)	0.0020 (8)	0.0022 (8)	0.0021 (8)
N1	0.0399 (9)	0.0357 (10)	0.0434 (10)	0.0052 (8)	0.0020 (8)	−0.0007 (8)
N3	0.0434 (10)	0.0372 (10)	0.0449 (10)	0.0043 (8)	0.0065 (8)	0.0070 (8)
N5	0.0473 (10)	0.0341 (10)	0.0486 (11)	−0.0012 (8)	0.0056 (8)	−0.0010 (8)
C27	0.132 (3)	0.0428 (16)	0.087 (2)	−0.0277 (18)	0.021 (2)	−0.0182 (16)
N4	0.0471 (10)	0.0395 (10)	0.0406 (10)	0.0087 (8)	0.0080 (8)	0.0085 (8)
C7	0.0463 (12)	0.0391 (12)	0.0395 (11)	0.0083 (10)	0.0026 (9)	−0.0033 (9)
C18	0.0390 (11)	0.0327 (11)	0.0421 (11)	0.0045 (9)	0.0043 (9)	0.0014 (9)
O1	0.0439 (9)	0.0694 (12)	0.0732 (13)	0.0254 (9)	−0.0082 (8)	−0.0251 (10)
O2	0.0709 (12)	0.0669 (12)	0.0464 (9)	0.0307 (10)	0.0216 (8)	0.0219 (9)
C19	0.0465 (12)	0.0367 (11)	0.0378 (11)	0.0112 (9)	0.0092 (9)	0.0038 (9)
C5	0.0432 (11)	0.0347 (11)	0.0400 (11)	0.0033 (9)	0.0012 (9)	0.0004 (9)
O3	0.0852 (13)	0.0419 (9)	0.0445 (9)	0.0005 (9)	−0.0032 (9)	0.0069 (7)
C17	0.0398 (11)	0.0352 (11)	0.0402 (11)	0.0009 (9)	0.0047 (9)	0.0012 (9)
C13	0.0487 (13)	0.0478 (14)	0.0557 (14)	0.0033 (11)	0.0073 (11)	0.0153 (11)
C31	0.0542 (14)	0.0535 (15)	0.0435 (13)	−0.0110 (12)	0.0077 (11)	−0.0074 (11)
C6	0.0438 (12)	0.0349 (11)	0.0412 (11)	0.0060 (9)	0.0044 (9)	−0.0029 (9)
C21	0.087 (2)	0.0373 (13)	0.0674 (17)	0.0171 (13)	0.0259 (16)	0.0089 (12)
C1	0.0426 (12)	0.0432 (13)	0.0523 (13)	0.0081 (10)	0.0033 (10)	−0.0012 (10)
C4	0.0551 (14)	0.0561 (15)	0.0441 (13)	0.0093 (12)	−0.0024 (11)	−0.0088 (11)
C30	0.0434 (12)	0.0454 (13)	0.0424 (12)	−0.0003 (10)	0.0028 (9)	−0.0039 (10)
C24	0.0435 (13)	0.0515 (14)	0.0597 (15)	0.0071 (11)	0.0062 (11)	0.0058 (12)
C14	0.0586 (15)	0.0644 (17)	0.0491 (14)	−0.0020 (13)	0.0124 (12)	0.0132 (12)
C25	0.0626 (15)	0.0399 (13)	0.0585 (15)	−0.0035 (11)	0.0066 (12)	0.0045 (11)
C20	0.0555 (14)	0.0422 (13)	0.0585 (15)	0.0070 (11)	0.0094 (12)	0.0036 (11)
C29	0.0498 (13)	0.0422 (13)	0.0497 (13)	−0.0042 (10)	0.0073 (11)	−0.0068 (10)
C23	0.0500 (14)	0.084 (2)	0.0590 (16)	0.0265 (14)	0.0082 (12)	0.0115 (15)
C32	0.0616 (16)	0.0540 (15)	0.0503 (14)	−0.0100 (12)	0.0015 (12)	−0.0005 (12)
C16	0.0565 (14)	0.0481 (14)	0.0521 (14)	0.0096 (11)	0.0131 (11)	0.0009 (11)
C33	0.086 (2)	0.077 (2)	0.0556 (17)	−0.0239 (18)	−0.0106 (16)	0.0101 (15)
C11	0.089 (2)	0.0368 (13)	0.0653 (17)	0.0058 (13)	0.0113 (15)	−0.0059 (12)
C12	0.0658 (16)	0.0435 (14)	0.0570 (15)	0.0007 (12)	0.0186 (12)	−0.0038 (11)
C2	0.0427 (13)	0.0603 (16)	0.0625 (16)	0.0095 (12)	−0.0041 (11)	0.0003 (13)
C22	0.085 (2)	0.0652 (19)	0.0611 (17)	0.0458 (17)	0.0277 (15)	0.0205 (14)
C8	0.0739 (18)	0.0513 (15)	0.0576 (16)	−0.0027 (13)	0.0204 (14)	−0.0051 (12)
C9	0.084 (2)	0.078 (2)	0.0618 (17)	0.0028 (17)	0.0327 (16)	−0.0137 (15)
C3	0.0541 (15)	0.0714 (18)	0.0550 (15)	0.0049 (13)	−0.0141 (12)	−0.0037 (13)
C15	0.0648 (16)	0.0647 (17)	0.0521 (15)	0.0055 (14)	0.0203 (13)	0.0008 (13)
C35	0.100 (3)	0.098 (3)	0.063 (2)	−0.018 (2)	0.0377 (19)	−0.0240 (18)
C26	0.094 (2)	0.0370 (14)	0.088 (2)	−0.0098 (14)	0.0204 (18)	0.0034 (14)
C10	0.080 (2)	0.0618 (18)	0.0586 (16)	0.0186 (15)	0.0136 (15)	−0.0186 (14)
C36	0.0677 (18)	0.082 (2)	0.0618 (17)	−0.0014 (16)	0.0121 (14)	−0.0123 (15)
C34	0.115 (3)	0.103 (3)	0.0442 (16)	−0.040 (2)	0.0093 (19)	−0.0032 (17)
C28	0.102 (2)	0.0538 (17)	0.0621 (17)	−0.0224 (16)	0.0136 (16)	−0.0154 (14)

### Geometric parameters (Å, º)

**Table:**

Nd1—N2	2.604 (2)	C21—H21	0.9300
Nd1—N1	2.661 (2)	C1—C2	1.377 (3)
Nd1—N5	2.680 (2)	C1—H1	0.9300
Nd1—N4	2.6953 (19)	C4—C3	1.384 (4)
Nd1—N6	2.7018 (19)	C4—H4	0.9300
Nd1—N3	2.742 (2)	C30—C29	1.485 (3)
Nd1—Cl3	2.7686 (8)	C24—C23	1.384 (4)
Nd1—Cl2	2.7903 (9)	C24—H24	0.9300
Nd1—Cl1	2.8296 (10)	C14—C15	1.372 (4)
N2—C6	1.277 (3)	C14—H14	0.9300
N2—O1	1.380 (2)	C25—C26	1.370 (4)
N6—C30	1.275 (3)	C25—H25	0.9300
N6—O3	1.387 (2)	C20—H20	0.9300
N1—C1	1.339 (3)	C29—C28	1.380 (4)
N1—C5	1.354 (3)	C23—C22	1.371 (4)
N3—C13	1.336 (3)	C23—H23	0.9300
N3—C17	1.355 (3)	C32—C33	1.374 (4)
N5—C25	1.339 (3)	C32—H32	0.9300
N5—C29	1.342 (3)	C16—C15	1.380 (4)
C27—C26	1.363 (5)	C16—H16	0.9300
C27—C28	1.385 (4)	C33—C34	1.369 (5)
C27—H27	0.9300	C33—H33	0.9300
N4—C18	1.278 (3)	C11—C10	1.358 (4)
N4—O2	1.383 (2)	C11—C12	1.390 (4)
C7—C12	1.372 (3)	C11—H11	0.9300
C7—C8	1.386 (3)	C12—H12	0.9300
C7—C6	1.481 (3)	C2—C3	1.365 (4)
C18—C17	1.479 (3)	C2—H2	0.9300
C18—C19	1.485 (3)	C22—H22	0.9300
O1—H1A	0.8200	C8—C9	1.378 (4)
O2—H2A	0.8200	C8—H8	0.9300
C19—C24	1.377 (3)	C9—C10	1.362 (4)
C19—C20	1.390 (3)	C9—H9	0.9300
C5—C4	1.381 (3)	C3—H3	0.9300
C5—C6	1.485 (3)	C15—H15	0.9300
O3—H3A	0.8200	C35—C34	1.367 (5)
C17—C16	1.376 (3)	C35—C36	1.388 (4)
C13—C14	1.375 (4)	C35—H35	0.9300
C13—H13	0.9300	C26—H26	0.9300
C31—C32	1.389 (4)	C10—H10	0.9300
C31—C36	1.389 (4)	C36—H36	0.9300
C31—C30	1.486 (3)	C34—H34	0.9300
C21—C22	1.366 (4)	C28—H28	0.9300
C21—C20	1.379 (4)		
			
N2—Nd1—N1	60.38 (6)	C7—C6—C5	120.62 (19)
N2—Nd1—N5	146.60 (6)	C22—C21—C20	120.3 (3)
N1—Nd1—N5	140.98 (6)	C22—C21—H21	119.9
N2—Nd1—N4	121.50 (6)	C20—C21—H21	119.9
N1—Nd1—N4	72.25 (6)	N1—C1—C2	123.5 (2)
N5—Nd1—N4	68.74 (6)	N1—C1—H1	118.3
N2—Nd1—N6	126.82 (6)	C2—C1—H1	118.3
N1—Nd1—N6	139.68 (6)	C5—C4—C3	119.1 (2)
N5—Nd1—N6	59.14 (6)	C5—C4—H4	120.4
N4—Nd1—N6	111.33 (6)	C3—C4—H4	120.4
N2—Nd1—N3	137.44 (6)	N6—C30—C29	115.7 (2)
N1—Nd1—N3	83.49 (6)	N6—C30—C31	123.3 (2)
N5—Nd1—N3	75.89 (6)	C29—C30—C31	121.0 (2)
N4—Nd1—N3	58.57 (6)	C19—C24—C23	119.5 (3)
N6—Nd1—N3	67.56 (6)	C19—C24—H24	120.3
N2—Nd1—Cl3	74.36 (5)	C23—C24—H24	120.3
N1—Nd1—Cl3	134.58 (4)	C15—C14—C13	118.6 (2)
N5—Nd1—Cl3	78.41 (5)	C15—C14—H14	120.7
N4—Nd1—Cl3	136.62 (5)	C13—C14—H14	120.7
N6—Nd1—Cl3	71.65 (5)	N5—C25—C26	123.7 (3)
N3—Nd1—Cl3	138.76 (4)	N5—C25—H25	118.1
N2—Nd1—Cl2	69.77 (5)	C26—C25—H25	118.1
N1—Nd1—Cl2	77.37 (5)	C21—C20—C19	119.4 (3)
N5—Nd1—Cl2	130.24 (4)	C21—C20—H20	120.3
N4—Nd1—Cl2	131.64 (4)	C19—C20—H20	120.3
N6—Nd1—Cl2	71.34 (5)	N5—C29—C28	121.7 (2)
N3—Nd1—Cl2	81.64 (5)	N5—C29—C30	117.4 (2)
Cl3—Nd1—Cl2	91.19 (4)	C28—C29—C30	120.9 (2)
N2—Nd1—Cl1	70.22 (5)	C22—C23—C24	120.2 (3)
N1—Nd1—Cl1	81.67 (5)	C22—C23—H23	119.9
N5—Nd1—Cl1	86.33 (5)	C24—C23—H23	119.9
N4—Nd1—Cl1	70.85 (5)	C33—C32—C31	120.5 (3)
N6—Nd1—Cl1	138.42 (5)	C33—C32—H32	119.8
N3—Nd1—Cl1	129.42 (4)	C31—C32—H32	119.8
Cl3—Nd1—Cl1	79.77 (3)	C17—C16—C15	119.1 (2)
Cl2—Nd1—Cl1	139.95 (3)	C17—C16—H16	120.5
C6—N2—O1	113.33 (18)	C15—C16—H16	120.5
C6—N2—Nd1	126.43 (14)	C34—C33—C32	119.9 (3)
O1—N2—Nd1	120.13 (13)	C34—C33—H33	120.0
C30—N6—O3	113.23 (18)	C32—C33—H33	120.0
C30—N6—Nd1	125.00 (15)	C10—C11—C12	120.1 (3)
O3—N6—Nd1	121.56 (13)	C10—C11—H11	120.0
C1—N1—C5	117.13 (19)	C12—C11—H11	120.0
C1—N1—Nd1	122.39 (15)	C7—C12—C11	120.2 (2)
C5—N1—Nd1	120.07 (14)	C7—C12—H12	119.9
C13—N3—C17	116.6 (2)	C11—C12—H12	119.9
C13—N3—Nd1	121.58 (15)	C3—C2—C1	119.0 (2)
C17—N3—Nd1	119.27 (14)	C3—C2—H2	120.5
C25—N5—C29	117.5 (2)	C1—C2—H2	120.5
C25—N5—Nd1	120.04 (16)	C21—C22—C23	120.4 (2)
C29—N5—Nd1	122.42 (15)	C21—C22—H22	119.8
C26—C27—C28	118.7 (3)	C23—C22—H22	119.8
C26—C27—H27	120.6	C9—C8—C7	120.3 (3)
C28—C27—H27	120.6	C9—C8—H8	119.8
C18—N4—O2	112.71 (17)	C7—C8—H8	119.8
C18—N4—Nd1	125.20 (14)	C10—C9—C8	120.1 (3)
O2—N4—Nd1	122.00 (12)	C10—C9—H9	120.0
C12—C7—C8	118.9 (2)	C8—C9—H9	120.0
C12—C7—C6	120.9 (2)	C2—C3—C4	119.0 (2)
C8—C7—C6	120.1 (2)	C2—C3—H3	120.5
N4—C18—C17	116.40 (18)	C4—C3—H3	120.5
N4—C18—C19	122.86 (19)	C14—C15—C16	118.9 (2)
C17—C18—C19	120.71 (18)	C14—C15—H15	120.6
N2—O1—H1A	109.5	C16—C15—H15	120.6
N4—O2—H2A	109.5	C34—C35—C36	120.1 (3)
C24—C19—C20	120.1 (2)	C34—C35—H35	120.0
C24—C19—C18	119.6 (2)	C36—C35—H35	120.0
C20—C19—C18	120.2 (2)	C27—C26—C25	118.7 (3)
N1—C5—C4	122.2 (2)	C27—C26—H26	120.6
N1—C5—C6	116.83 (19)	C25—C26—H26	120.6
C4—C5—C6	120.9 (2)	C11—C10—C9	120.5 (3)
N6—O3—H3A	109.5	C11—C10—H10	119.8
N3—C17—C16	122.7 (2)	C9—C10—H10	119.8
N3—C17—C18	116.38 (19)	C35—C36—C31	119.7 (3)
C16—C17—C18	120.9 (2)	C35—C36—H36	120.2
N3—C13—C14	124.0 (2)	C31—C36—H36	120.2
N3—C13—H13	118.0	C35—C34—C33	120.7 (3)
C14—C13—H13	118.0	C35—C34—H34	119.7
C32—C31—C36	119.1 (2)	C33—C34—H34	119.7
C32—C31—C30	120.8 (2)	C29—C28—C27	119.5 (3)
C36—C31—C30	120.1 (3)	C29—C28—H28	120.2
N2—C6—C7	124.1 (2)	C27—C28—H28	120.2
N2—C6—C5	115.26 (19)		

### Hydrogen-bond geometry (Å, º)

**Table:**

*D*—H···*A*	*D*—H	H···*A*	*D*···*A*	*D*—H···*A*
O1—H1*A*···Cl3	0.82	2.22	2.966 (2)	152
O2—H2*A*···Cl1	0.82	2.19	2.9290 (19)	151
O3—H3*A*···Cl2	0.82	2.19	2.930 (2)	150
